# Fruits By-Products – A Source of Valuable Active Principles. A Short Review

**DOI:** 10.3389/fbioe.2020.00319

**Published:** 2020-04-15

**Authors:** Radu C. Fierascu, Elwira Sieniawska, Alina Ortan, Irina Fierascu, Jianbo Xiao

**Affiliations:** ^1^University of Agronomic Sciences and Veterinary Medicine of Bucharest, Bucharest, Romania; ^2^The National Institute for Research & Development in Chemistry and Petrochemistry-ICECHIM, Emerging Nanotechnologies Group, Bucharest, Romania; ^3^Department of Pharmacognosy, Medical University of Lublin, Lublin, Poland; ^4^International Research Center for Food Nutrition and Safety, Jiangsu University, Zhenjiang, China

**Keywords:** fruits waste, fruits processing, active compounds, value added products, therapeutic properties, side streams

## Abstract

The growing demand for more sustainable, alternative processes leading to production of plant-derived preparations imposes the use of plants waste generated mainly by agri-food and pharmaceutical industries. These mostly unexploited but large quantities of plants waste also increase the interest in developing alternative approaches for sustainable production of therapeutic molecules. In order to reduce the amount of plant waste by further processing, different novel extraction techniques can be applied. Fruits and their industrial by-products are rich sources of different classes of compounds with therapeutic properties. The processed fruits waste can be reused and lead to novel pharmaceuticals, food supplements or functional foods. This review intends to briefly summarize recent aspects regarding the production of different active compounds from fruit by-products, and their therapeutic properties. The potential use of fruits by-products in different industries will be also discussed.

## Introduction

According to the United Nation’s Food and Agriculture Organization, the production of different fruits around the world exceeded one billion tones in 2017, which, depending on geographical areas, consumption and growing traditions, inevitably leads to the generation of large amounts of by-products and waste (Food and Agriculture Organization of the United Nations [FAO], 2017). Only Europe generates approximately 100 Mt of waste and by-products each year, being an outcome of drinks industry (26%), as well as fruits and vegetables companies (14.8%) (Marić et al., 2018). The management of by-products resulted from fruits processing is one of the major problems of agri-food industries. Large quantities of waste containing a rich composition of biological compounds can be considered hazardous as may cause phytotoxicity phenomena, including plant growth interference, contamination of the aqueous media, deterioration of drinking water quality, death of sensitive marine organisms, inhibition of seed germination, and intestinal disorders in fed animals (Klapa, 2015). Even if these large amounts of waste can lead to environmental issues, at the same time, with a proper treatment, they can represent a low-cost raw material, rich in potentially valuable components for other industries (Rabetafika et al., 2014). Applying various extraction and purification techniques and analytical methods, it is possible to recover important bioactive components from fruits waste and further transform them into added value products for other industries (Nile et al., 2019). Removal, proper disposal and bio-waste processing can lead to the closure of bio-economical value chains, providing the opportunity to reduce serious environmental concerns regarding food waste.

The most common fruits consumed worldwide are apples, grapes, and exotic fruits typical for the cultivation region. Apples are the most popular fruits that provide production of juice and cider production in over three million tons in Europe (Barreira et al., 2019) and about 5 million tons in United States each year (Niglio et al., 2019). The remaining residual material after processing is pomace, which consists of peels, skin, pulp, leftover flesh, core with seeds and stems, being rich in polysaccharides, phenolic compounds, and phenolic acids (Coman et al., 2019). Apple pomace contains larger amounts of bioactive substances compared to the juices from the fruit itself (Michalska and Łysiak, 2014).

According to international statistics, grapes are world’s largest fruit crop, with an annual production of over 75 million of tons worldwide (International Organisation of Vine and Wine [OIV], 2017). In this context, grapes and products obtained, such as wine, grape juice, jams, have an obvious economic importance and a considerable impact on production of waste. The marc, peels and grape seeds resulting from wine industry remain deposited due to the lack of a proper management, thus being a source of contamination. On the other hand, grape waste represents a good source of vitamins, minerals, lipids, proteins, carbohydrates and polyphenolic compounds (especially phenolic acids, ellagitannins, flavonols, flavan-3-ols such as catechins and their isomers, anthocyanins, pro-anthocyanidins and the stilbene derivative, resveratrol) (Nowshehri et al., 2015). Grape wastes are not considered hazardous waste, but in large amounts they can contribute to the pollution, due to the high chemical and biological oxygen demand (Martinez et al., 2016).

Other fruits with a large production are the exotic fruits like coffee, macadamia, pineapple, papaya, and mango. They are rich in vitamins, carotenoids, phenolic compounds and dietary fiber distributed in different parts of the fruits (pericarp, peels or seeds) (Ayala-Zavala et al., 2011).

In this context, our short review intends to present a critical opinion on aspects regarding the production of active compounds from different fruits by-products, their therapeutic properties and the potential use of fruit by-products in different industries, being a link for researchers from different sciences: chemistry, pharmacology and medicine.

## Production of Active Principles From Fruit By-Products

Scientific development registered in the last decades in the field of extraction of biologically active molecules enables the valorization and consequently utilization of fruit processing by-products. Thousands of valuable molecules generated from fruits waste can be used in food, cosmetic, or pharma industry (Mourtzinos and Goula, 2019). In order to obtain added-value products, extraction methods have to be elaborated for each side-product generated from agro-food industries (Bustamante et al., 2008). Traditional methods are still in use, even if they cause high consumption of energy and sometimes degradation of thermolabile compounds. Sustainable methods are continuously developed by improving and optimizing the existing processes following the principles of green chemistry (Table 1).

**TABLE 1:** Different classes of compounds extracted from fruit by-products and their potential industrial application.

Fruit	Fruit by-product	Active principle	Extraction method	Reference	Industrial application	Reference
Grape	Grape marc, skin, pomace, seeds	Polyphenols	Supercritical fluid extraction; microwave extraction; ultrasound-assisted extraction; enzyme-aided extraction; pulsed electric fields processing; high voltage electrical discharges	Kelly et al., 2019	Food industry	Andrade et al., 2019
	Wine less	Polyphenols	Classical extraction with microwave and ultrasounds as pre-treatments	Romero-Díez et al., 2019	Pharmaceutic, cosmetic industry and biofuel production	Banerjee et al., 2017
	Grape marc	Pectins	Ultrasound-assisted extraction	Minjares-Fuentes et al., 2014		
	Skin	Phenolic compounds	Natural deep eutectic solvents (NADES) ultrasonic extraction	Radosevic et al., 2016		
	Pomace	Anthocyanins	Solid liquid extraction using ionic liquid solutions	Lima et al., 2017		
	Grape marc	Monomeric anthocyanins, phenolic compounds	Pressurized liquid extraction	Pereira et al., 2019		
	Seeds	Phenolic compounds, anthocyanins	Ultrasound-assisted extraction	Ghafoor et al., 2009		
	Pomace	Phenolic compounds	Enzymatic extraction process	Meini et al., 2019	Beverage industry	Reissner et al., 2019
	Pomace	Anthocyanins	Enzymatic extraction process using brewery’s yeast biomass	Stafussa et al., 2016		
	Winery grape waste	Cellulose, hemicelluloses and lignin	Enzymatic extraction process	Karpe et al., 2014		
	Pomace, seeds, skins and stems	Phenolic compounds	High voltage electrical discharges	Boussetta et al., 2012		
	Pomace	Pullulan	Submerged fermentation	Singh et al., 2019	Packaging materials	Singh et al., 2019
Coffee	Spent ground coffee	Phenolic compounds	High voltage electrical discharges	Boussetta et al., 2012	Fructooligosaccharides production	de la Rosa et al., 2019
Apple	Pomace	Phenolic compounds	Supercritical fluid extraction	Ferrentino et al., 2018	Production of microbial oils, application as biofuel	Liu et al., 2019
					Cosmetic industry	Barreira et al., 2019
					Bioethanol production	Evcan and Tari, 2015
Mango	Peels	Carotenoids, phenolics and flavonoids	Supercritical CO_2_ extraction followed by pressurized ethanol from the residue of the first stage	Garcia-Mendoza et al., 2015	Food and pharmaceutical industry	Ruiz-Montañez et al., 2014
	Peels	Phenolic compounds	Pulsed electric fields; high voltage electrical discharges	Parniakov et al., 2015		
Orange	Peels	Essential oil, polyphenols and pectin	Ultrasound and microwave extraction	Boukroufa et al., 2015	Fructooligosaccharides production	de la Rosa et al., 2019
Orange	Peels	Ferulic acid	Solid liquid extraction using deep eutectic solvents	Ozturk et al., 2018		

The most common target compounds from fruit by-products are polyphenols present in skins, pulp, seeds, or pomace (Kelly et al., 2019). They are unstable in high temperatures, which significantly reduces their concentration levels, therefore extraction at elevated temperatures is not recommended for these compounds. Hence, classical solid-liquid extraction techniques should be replaced with the *state-of-the-art* techniques. However, in this case, equipment needed and optimization parameters are important issues for obtaining active compounds from by-products. The most suitable and frequently used for isolation of polyphenolic compounds from fruits by-products are extraction techniques involving pressure, microwave and ultrasounds. In order to increase the yield of bioactive compounds and decrease amount of solvents used, and energy consumption, extraction techniques usually involve some pre-treatment and post-treatment processes. Pre-treatment processes are typically applied to remove lignin, reduce cellulose crystallinity and increase cell porosity (Kumar et al., 2009). Polyphenols non-covalently interact with polysaccharides and become non-extractable by polar solvents (Pérez-Jiménez et al., 2013). Pre-treatment releases polyphenols from the matrix and determines higher yields. When Romero-Díez et al. (2019) performed conventional solid-liquid extraction preceded by microwaves (MW) and ultrasounds (US) pre-treatments, the yield of anthocyanins from wine lees was doubled.

As stated before, the application of modern extraction techniques results in increased yields of different active compounds (Minjares-Fuentes et al., 2014; Manna et al., 2015; Ferrentino et al., 2018; Zachová et al., 2018; Pereira et al., 2019). The advantages of pressurized liquid extraction (super and sub-critical fluid extraction) are low processing temperature (removing the possibility of thermal degradation of phytochemicals), the ease of separation with no solvent residue in the recovered substance, and the minimization of undesirable oxidation reactions. However, this technique presents a major disadvantage, as it can be applied only for isolation of compounds of low or medium polarity (Garcia-Salas et al., 2010). In microwave assisted techniques, electromagnetic radiation causes the rupture of cell walls in plant material in a short time. By applying a solvent that absorbs microwaves energy and has a good affinity with extracted compounds, an increased polyphenols yield can be easily obtained. The disadvantages of this method are the inflammability of the solvents and the multitude of parameters to be optimized (Garcia-Salas et al., 2010). Ultrasound assisted techniques are also advantageous, requiring lower temperatures, lower quantities of solvents, and favoring the solubilization of the targeted compounds. However, the formation of free radicals during extraction process is a drawback which should be mentioned (Ghafoor et al., 2009). The methods described above are also adequate to be used for recovery of compounds such as pigments (Garcia-Mendoza et al., 2015), essential oils or pectins (Boukroufa et al., 2015).

Other green extraction method includes the application of enzymes or acids. The main benefit of enzymatic extraction is the shorter processing time, minimization of organic solvent used, increased purity and larger quantity of bioactive components. This method is appropriate for increasing the extractability of polyphenols, covering usually non-extractable proanthocyanidins (Radenkovs et al., 2018). Meini et al. (2019) demonstrated the positive effect of pectinase, cellulase and tannase on the release of phenolics from grape pomace matrix, while Stafussa et al. (2016) investigated the possibility to inter-connect two food industry by-products, using brewery’s yeast biomass as biosorbent of the anthocyanins from grape pomace. Also, various fungi such as *Trichoderma*, *Aspergillus*, and *Penicillium* sp. have been reported as biomass degraders due to their property to generate an array of enzymes such as endo- and exo-glucanases, β-glucosidase, xylanases, arabinofuranosidases and pectinases, obtaining cellulose, hemicelluloses and lignin (Karpe et al., 2014).

In the last decades, innovative technologies, like Pulsed Electric Fields (PEF), High Voltage Electrical Discharges (HVED) or Pulsed Ohmic Heating (POH) have been investigated, mostly for the recovery of phenolic compounds related to valorization of fruits wastes and by-products. PEF enabled to obtain high content of polyphenols from vine shoots (conventionally used as a heating source or left on the ground to rot) (Rajha et al., 2014). HVED provided the intensification of the extraction of total polyphenols from grape pomace, seeds, skins and stems, resulting in 7 times increased yield at both laboratory and pilot scale (Boussetta et al., 2012). The application of techniques involving electric energy allowed the selective recovery of valuable compounds from different fruits waste in a sustainable, economical, and environmentally friendly way (Parniakov et al., 2015).

The important challenge in the recovery of phenolic compounds from fruits waste is the choice of the solvent used, which must meet the REACH characteristics (Registration, Evaluation, Authorization and Restriction of Chemicals regulation) (Renard, 2018). Bio-based solvents, subcritical liquids, ionic liquids or (natural) deep eutectic solvents are now often used (Radosevic et al., 2016; Lima et al., 2017; Ozturk et al., 2018). These modern solvents present some advantages such as non-flammability, low toxicity, biocompatibility and the possibility to be obtained from renewable materials. Their properties can be tuned by modifying the hydrogen bond acceptor/donor structures or by changing the molar ratio of their components (Ozturk et al., 2018). Using these solvents, the yield of recovery of phenolic compounds can be enhanced by increasing the operating temperature without damaging active principles, due to the increased solubility and diffusion coefficients of polyphenolic compounds in the solvents. Their polarity can be changed by modifying their composition, so they can be used to solubilize a wide variety of bioactive compounds (Benvenutti et al., 2019).

The economic feasibility of the extraction method depends on yield of recovery of active compounds. Due to the diversity of constituents present in fruit waste, a fractionation strategy would improve the efficiency and cost of the processes. When the process involves the recovery of several compounds from fruits by-products, a major concern to consider is the order of extraction. Due to mechanic processes like pressing, phenolic compounds may bind to cell wall, leading to decreased extraction yield. This interaction reduces also the extractability of pectins. Therefore, in any given process designed to obtain more than phenolic compounds, these should be extracted first. Moreover, the fact that the extraction process is time and solvent consuming, the order of extraction of the compounds can be the bottleneck of the entire biorefinery process (Perussello et al., 2017).

## Therapeutic Properties of Active Compounds From Fruits By-Products

Therapeutic properties of bioactive compounds derived from fruits are widely described in the scientific literature. A number of reviews summarized anticancer, antidiabetic, antihypertensive, anti-inflammatory, antimicrobial, antioxidant, immunomodulatory, or neuroprotective activity of plant secondary metabolites extracted from fruits, underlining their importance in the human diet (Banerjee et al., 2017; Yalcin and Çapar, 2017; Karasawa and Mohan, 2018; Marli et al., 2018; van Breda and de Kok, 2018; Fidelis et al., 2019). Scientific information related to biological activities of raw fruits by-products is focused mainly on food applications. Antioxidant and antimicrobial properties of seeds, flesh, peels, or pulp waste are investigated in order to design novel functional foods enhancing human health and well-being (Park et al., 2014; Chougui et al., 2015; Chen et al., 2017; Zafra-Rojas et al., 2018; Saleem and Saeed, 2020). Since fruits by-products are mainly the outcome of food industry, the application of food waste within the same industry ensures circulation of the redundant food biomass and facilitates the waste management. Nevertheless, regardless the benefits which are obtained by processing fruits by-products within food industry, current preliminary scientific evidence confirms that fruits waste can be considered as a valuable source of phytoconstituents for medicinal applications. Anti-inflammatory potential of avocado peel and seed extracts was reported to suppress the release of tumor necrosis factor-α and generation of nitric oxide in lipopolysaccharide-stimulated RAW264.7 macrophages (Tremocoldi et al., 2018). Gastroprotective activity was reported for avocado seeds extract against indomethacin-induced gastric ulcer in mice (Athaydes et al., 2019). Polyphenols extracted from orange flesh and peel were shown to protect human leukocytes against oxidative DNA damage and HepG2 cells against peroxyl radical-induced oxidation *in vitro* (Park et al., 2014). Orange peel polyphenols had also the ability to inhibit the activity of inducible nitric oxide synthase and cyclooxygenase-2 in murine macrophage cell line (Chen et al., 2017). Ellagitannins, ellagic acid and its metabolites (urolithin A and urolithin B) isolated from black raspberry seeds, a major waste product after winemaking, showed anti-cancer activities against HT-29 colon cancer cells. Investigated compounds arrested the cell cycle and induced extrinsic and intrinsic apoptotic pathways (Cho et al., 2015). Pomegranate peel extract prevented bone loss in a mice model of osteoporosis and stimulated osteoblastic differentiation *in vitro* (Spilmont et al., 2015). Aromatic glycosides obtained from *Prunus tomentosa* seed waste inhibited α-glucosidase at the concentration level comparable to positive control acarbose and exhibited significant protective effect against H2O2-induced HepG2 cells damage (Liu et al., 2018). Catechins present in the grape seeds showed potential to inhibit angiotensin-I-converting enzyme *in vitro* (Deolindo et al., 2019), while anti-inflammatory effect of grape seed extract in LPS-induced RAW264.7 macrophages was attributed to flavonoids (Harbeoui et al., 2019). Also, pomegranate seed oil positively influenced fatty acids profile and reduced the activity of desaturases in rat’s liver (Białek et al., 2017).

High biological potential of redundant fruit biomass can be utilized by pharmaceutical industry. However, processing of fruits waste for possible medicinal applications requires strict standardization of extracts or determination of purity of isolated compounds. What is more, before ingredients of fruits waste can be applied in a form of drugs, preliminary *in vitro* studies have to be confirmed not only in animal models, but also in clinical trials. Anyhow, standardized extracts or isolated active compounds incorporated into dietary supplements or functional cosmetics can also be a stream for fruits waste management. The re-utilization of food by-products is the easy way for sustainable production of active compounds and this idea gained recently a lot of interest. With the application of efficient extraction techniques fruits by-products can become a cheap source of active compounds.

## Industrial Applications of Valuable Compounds Obtained From Fruits By-Products

Large amounts of wastes and by-products are produced at industrial levels, most of them containing highly valuable bioactive substances, which can be further used for different applications (Table 1). Hence, another scientific area is emerging within the large field of food science and technology – bio-residues valorization (Martins and Ferreira, 2017). Fruits waste can be used as such for some applications or for recovery of valuable compounds with therapeutic properties.

Several applications of compounds recovered from different industries are well known. Compounds recovered from fruits wastes find further applications as additives in food products to preserve and enhance quality, to prevent food oxidation and to inhibit the growth of pathogenic microorganisms (Andrade et al., 2019). Also, added-value products obtained from fruits wastes can be used as novel packaging materials due to their oxygen-impermeable properties (Singh and Kaur, 2015) or as functional co-products (Nagel et al., 2014). Fruits wastes can be used in food industry as a substitute of wheat flour (Struck et al., 2018), can be added in cakes (Tumbas Šaponjac et al., 2016) or can be used in beverage industry (Reissner et al., 2019). The interest in natural-based products was expanded also in cosmetic industry, which is a profitable solution to valorize disposable by-products. Search for new drugs and increased resistance of the actual ones led to finding new sources of antimicrobial agents, such as recovered bioactive compounds from fruits waste (Ruiz-Montañez et al., 2014). In the last decades, the interest in using biomass for the production of energy has become an important strategy aimed to reduce the negative environmental impact of fossil fuels. In this context, value added products can be produced by fermentation of different wastes by anaerobic bacteria, which can be integrated into a fed-batch or continuous system for lipid or biofuel production (Liu et al., 2019).

Due to rich composition in sucrose, inulin, maltose, glucose, fructose, starch, galactose, dextrose or lactose, fruits waste can be used as substrate for production of oligosaccharides or microbial polymers (Singh et al., 2019). Conventionally, polysaccharides are produced using sucrose as substrate, and enzymes such as β-fructofuranosidase and fructosyltransferase (de la Rosa et al., 2019). However, nowadays, more profitable processes involve fruits by-products and microorganisms such as *Aspergillus versicolor* (Dapper et al., 2016) or *Aspergillus flavus* (Ganaie et al., 2017). The agro industrial wastes which can be exploited for this purpose are those rich in sucrose: peels (agave mango, banana, pineapple, orange, etc.), leaves (banana, etc.), or pomaces (apple grape pomace) (Gnaneshwar Goud et al., 2013; Dapper et al., 2016). Fruits pomace can be also used to obtain pectin (used both in cosmetic and in food industry) (Barreira et al., 2019) or bioethanol, hydrogen or methane via anaerobic fermentation (Evcan and Tari, 2015). Most interesting applications of fruits waste are those which involve the cascade approaches of transforming recovered compounds into other different compounds. Compounds from fruits by-products (such as cellulose or hemicellulose) can be converted into sugars, further used for the production of biofuels and biochemicals or used for applications such as catalysis, chemical sensors and molecular separation (Banerjee et al., 2017).

## Conclusion

Fruits by-products can be considered a rich source for the recovery and production of multiple co-products in an integrated biorefinery model where green methods can be combined in order to obtain added-value products. The method used for the recovery of active constituents and the selection of the appropriate solvent have a great influence on the extractability of the functional compounds, no unique extraction procedure being suitable for all samples and matrixes.

Valuable compounds can be directly formulated as nutraceuticals for their proven health benefits or can be used as raw materials for other industries. The concept of recovery of different compounds from these wastes opens new routes for the development of “green” industries, which have a tremendous potential, especially where the availability of fruits waste is abundant. The emphasis on frontier sciences has already attracted interest in developing and optimizing new ecological methods for the efficient use of the biomass, for closing the chain by returning nutrients and organic matter to the soil, when all other useful products have been recovered.

## Author Contributions

RF, IF, and ES contributed to data collection and analysis, manuscript design and preparation. AO and JX revised the manuscript.

## Conflict of Interest

The authors declare that the research was conducted in the absence of any commercial or financial relationships that could be construed as a potential conflict of interest.
